# Growth hormone‐releasing hormone deficiency confers extended lifespan and metabolic resilience during high‐fat feeding in mid and late life

**DOI:** 10.1111/acel.14238

**Published:** 2024-06-12

**Authors:** Joseph Adkins‐Jablonsky, Alexander Tate Lasher, Amit Patki, Akash Nagarajan, Liou Y. Sun

**Affiliations:** ^1^ Department of Biology University of Alabama at Birmingham Birmingham Alabama USA; ^2^ Department of Biostatistics University of Alabama at Birmingham Birmingham Alabama USA

**Keywords:** aging, growth hormone‐releasing hormone, high fat diet, insulin, longevity

## Abstract

Growth hormone‐releasing hormone‐deficient (GHRH‐KO) mice have previously been characterized by lower body weight, disproportionately high body fat accumulation, preferential metabolism of lipids compared to carbohydrates, improved insulin sensitivity, and an extended lifespan. That these mice are long‐lived and insulin‐sensitive conflicts with the notion that adipose tissue accumulation drives the health detriments associated with obesity (i.e., diabetes), and indicates that GH signaling may be necessary for the development of adverse effects linked to obesity. This prompts investigation into the ultimate effect of diet‐induced obesity on the lifespan of these long‐lived mice. To this end, we initiated high‐fat feeding in mid and late‐life in GHRH‐KO and wild‐type (WT) mice. We carried out extensive lifespan analysis coupled with glucose/insulin tolerance testing and indirect calorimetry to gauge the metabolic effect of high‐fat dietary stress through adulthood on these mice. We show that under high‐fat diet (HFD) conditions, GHRH‐KO mice display extended lifespans relative to WT controls. We also show that GHRH‐KO mice are more insulin‐sensitive and display less dramatic changes in their metabolism relative to WT mice, with GHRH‐KO mice fed HFD displaying respiratory exchange ratios and glucose oxidation rates comparable to control‐diet fed GHRH‐KO mice, while WT mice fed HFD showed significant reductions in these parameters. Our results indicate that GH deficiency protects against the adverse effects of diet‐induced obesity in later life.

## Graphical abstract

Upon a high‐fat diet, long‐lived GHRH‐KO mice retain insulin sensitivity and consistent metabolic rates, contrary to wild‐type mice, indicating growth hormone deficiency may offer protection against obesity‐related metabolic impairment.

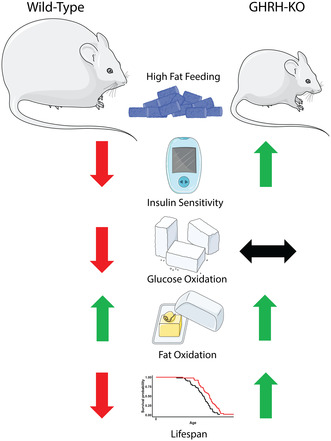

**Abbreviations**
CD: control diet
GH: growth hormone
GHR: growth hormone receptor
GHRH: growth hormone‐releasing hormone
HFD: high fat diet
IGF‐I: insulin‐like growth factor I
IL‐6: Interleukin 6
IPGTT: intraperitoneal glucose tolerance test
IPITT: intraperitoneal insulin tolerance test
PAPP‐A: pregnancy‐associated plasma protein‐A
RER: respiratory exchange ratio
VCO2: carbon dioxide production
VO2: oxygen consumption
WT: wild type

## 1 INTRODUCTION

Aging can broadly be defined as the gradual decline in an organism's physiological function as time advances. This progressive functional decline not only ultimately results in death but also positions age as a critical risk factor for many chronic health conditions such as type 2 diabetes, Alzheimer's disease, and cancer. Of these chronic conditions, obesity sits at a unique crossroads as it is also a risk factor for several diseases associated with advanced age and has become more prevalent in aged populations (Hales et al., 2018). Given that the aged population is ever‐growing in developed countries (Partridge et al., 2018), close study of obesity through advanced age is warranted to develop a more complete understanding of the biology of aging.

Genetic disruption of the growth hormone (GH) signaling pathway is among the most reproducible methods for lifespan extension in laboratory mice. Hypopituitary Ames and Snell dwarf mice, deficient in GH and other pituitary‐derived hormones, display significantly extended lifespans compared to their normal littermates (Brown‐Borg et al., 1996; Flurkey et al., 2001, 2002). Similar longevity enhancements have been reported in GH receptor (GHR) knockout mice (Coschigano et al., 2000), GH‐releasing hormone (GHRH) knockout (GHRH‐KO) mice (Sun et al., 2013), and mice with a double knockout of the GHR and GHRH genes (Icyuz et al., 2021). The commonality of this observation across several institutions and mouse genetic backgrounds highlights the importance of GH action in the aging process. We have previously shown that calorie restriction, a dietary intervention that has long been reported to extend rodent lifespan (Masoro, 2005; McCay et al., 1935; Swindell, 2012), further extends lifespan in GHRH‐KO mice (Sun et al., 2013). This suggests that the overall influence of dietary changes on lifespan works through different mechanisms than disrupted GH signaling. This prompts further exploration into how diet affects this model of extended lifespan.

In addition to their extended lifespan, GHRH‐KO mice are characterized by reduced body size, enhanced insulin sensitivity, greater adiposity, and elevated lipid metabolism relative to carbohydrates (Icyuz et al., 2020; Sun et al., 2013; Zhang et al., 2020). The physiological qualities of these healthy‐aging mice, particularly the increased lifespan and insulin sensitivity despite greater adipose accumulation, are at odds with the current understanding that adipose accumulation is a driver for insulin resistance/type 2 diabetes as well as other detriments (Ahmed et al., 2021). Previous research has shown that Ames dwarf mice are resistant to the insulin insensitivity and inflammation induced by high‐fat dietary stress despite disproportionate elevations in white adipose accumulation after body weight correction (Hill et al., 2016). This implies that some of the adverse effects of a high‐fat diet are, to some extent, dependent on functional GH signaling and that GH deficiency protects against the challenges posed by high dietary fat intake; however, some important questions remain unanswered. First, the impact of high‐fat feeding on the lifespan of GH‐deficient mice remains unknown. Second, the existing literature predominantly focuses on short‐term, early‐stage high‐fat feeding protocol, while leaving the impact of long‐term, late‐life dietary interventions largely unexplored. Finally, it remains uncertain whether GH inhibition alone directly contributes to protection from high‐fat diets, as this intervention hasn't been tested in a model solely featuring GH deficiency without other hormonal influences.

In this study, we investigate the influence of prolonged high‐fat diet consumption commenced in the mid or late stages of life, and continued until the end of life, in GH‐deficient mice. We also employ glucose tolerance testing, insulin tolerance testing, and indirect calorimetry to gauge metabolic changes that accompany this dietary intervention.

## 2 RESULTS

**FIGURE 1:**
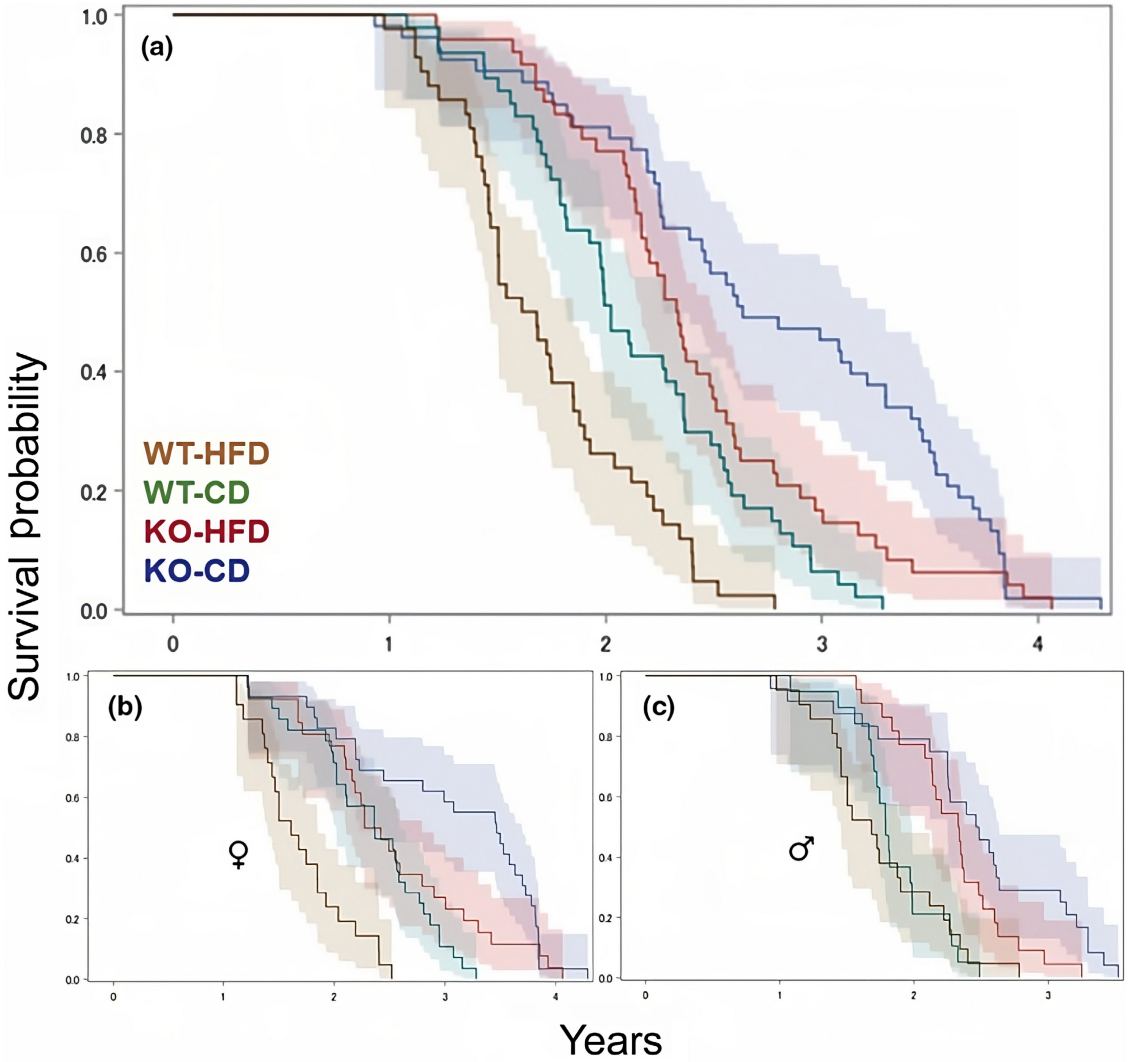
Effects of high‐fat diet on longevity. Pooled male and female mouse survival for wild‐type (WT) and growth hormone‐releasing hormone knockout (GHRH‐KO) mice fed high‐fat diet (HFD) or control diet (CD) (a). Survival analysis of female mice (b), and male mice (c). Shaded regions represent 95% CI. Sample sizes, statistical analyses employed, and *p*‐values are provided in Table 1.

To evaluate the effect of dietary stress in the form of high‐fat diet (HFD) onset in adulthood, we examined the survival of wild‐type (WT) and GHRH‐KO male and female mice maintained on a standard control diet (CD) or switched to HFD at 9‐months‐old. WT mice switched to HFD displayed significantly shorter median lifespans compared to WT mice maintained on CD, with the median lifespan being reduced from 2.02 years in the WT + CD mice to 1.65 in the WT + HFD mice (*p* = 0.0001; log‐rank test, Figure 1a). In GHRH‐KO mice the group switched to HFD displayed a reduced median lifespan of 2.33 years compared to the GHRH‐KO + CD group's median lifespan of 2.64 years (*p* = 0.0244; log‐rank test, Figure 1a). The lifespan of WT mice switched to HFD was significantly shortened compared to GHRH‐KO mice switched to HFD, with the WT + HFD and GHRH‐KO + HFD median lifespans being 1.65 and 2.33 years, respectively (*p* < 0.0001; log‐rank test, Figure 1a). To more completely study the survival of our cohort, we analyzed the sexes separately. Female WT mice switched to HFD displayed a median lifespan of 1.61 years, significantly reduced from the 2.36‐year median lifespan observed in WT female mice on CD (*p* < 0.0001; log‐rank test; Figure 1b). GHRH‐KO females fed HFD displayed a median lifespan of 2.35 years which was shorter than the 3.47‐year median lifespan of GHRH‐KO females fed CD but failed to reach statistical significance when analyzed by a log‐rank test (Figure 1b). Female WT mice switched to HFD displayed a significantly shorter median lifespan compared to female GHRH‐KO mice (1.61 years and 2.35 years, respectively; *p* < 0.0001; log‐rank test, Figure 1b). WT males switched to HFD had a median lifespan of 1.68 years, which was comparable to the CD‐fed WT male median lifespan of 1.79 years (Figure 1c). A similar pattern was observed in GHRH‐KO males, with GHRH‐KO + HFD males displaying a median lifespan of 2.33 years and GHRH‐KO + CD males displaying a median lifespan of 2.47 years, which trended toward but did not reach statistical significance (*p* = 0.0622; log‐rank test, Figure 1c). While no significant differences were detected within the same genotype for males, WT males fed HFD displayed a significantly shorter median lifespan compared to GHRH‐KO males fed HFD (1.68 years and 2.33 years, respectively; *p* = 0.0020; log‐rank test, Figure 1c).

A quantile regression method was employed to assess maximal longevity by comparing the age at which only upper (75th or 90th) percentiles of mice remained alive as previously published (Wang et al., 2004). WT mice switched to HFD displayed significantly decreased 75th percentile survival but not 90th percentile survival (*p* = 0.0479 and *p* = 0.7173, respectively: Table 1). This pattern was also observed in GHRH‐KO, with significantly reduced survival seen at the 75th percentile but not the 90th percentile (*p* = 0.0182 and *p* = 0.4925, respectively; Table 1). When the maximal lifespans of WT and GHRH‐KO mice fed HFD were compared, GHRH‐KO mice displayed significantly greater survival at the 75th and 90th percentiles over WT mice (*p* < 0.0001 both; Table 1). The small sample size that resulted when the sexes were separated prevented meaningful statistical analysis at the 75th and 90th percentiles. Quantile regression analysis for survival at the 50th percentile was consistent with the analysis of median lifespan reported above except for female GHRH‐KO mice fed HFD displaying significantly reduced survival at this percentile compared to GHRH‐KO females fed CD (*p* = 0.0315; Table 1).

**TABLE 1:** Statistical analysis of the lifespan of GHRH‐knockout and WT mice fed a high‐fat or control diet.

Gene	Gender	Diet	Number	Median lifespan	Wang–Allison *p*‐value*			Cox proportional Hazard		Log‐rank *p*‐value	Linear model	
					50th percentile	75th percentile	90th percentile	Hazard ratio	*p*‐Value		Adjusted mean lifespan	*p*‐Value
WT	All	HFD	42	1.65	0.0028	0.0479	0.7173	2.032	0.0028	0.0001	1.72	0.0004
		CD	47	2.02				1			2.11	
	Males	HFD	21	1.68	0.2049			1.093	0.7835	0.7825	1.75	0.4434
		CD	19	1.79				1			1.85	
	Females	HFD	21	1.61	0.0087			3.988	<0.0001	<0.0001	1.70	0.0001
		CD	28	2.36				1			2.32	
KO	All	HFD	48	2.33	0.017	0.0182	0.4925	1.61	0.0217	0.0244	2.39	0.0224
		CD	53	2.64				1			2.74	
	Males	HFD	22	2.33	0.3762			1.795	0.0655	0.0622	2.29	0.3918
		CD	24	2.47				1			2.44	
	Females	HFD	26	2.35	0.0315			1.584	0.1028	0.0971	2.51	0.0314
		CD	29	3.47				1			3.02	

Diet	Gender	Diet	Number	Median lifespan	Wang‐Allison *p*‐value*			Cox proportional Hazard		Log‐rank *p*‐value	Linear model	
					50th percentile	75th percentile	90th percentile	Hazard ratio	*p*‐Value		Adjusted mean lifespan	*p*‐Value
HFD	All	WT	42	1.65	<0.0001	<0.0001	<0.0001	1	<0.0001	<0.0001	1.72	<0.0001
		KO	48	2.33				0.297			2.41	
	Males	WT	21	1.68	<0.0001			1	0.0028	0.0020	1.75	0.0002
		KO	22	2.33				0.236			2.29	
	Females	WT	21	1.61	0.0006			1	<0.0001	<0.0001	1.70	<0.0001
		KO	26	2.35				0.383			2.51	

* Due to small sample sizes Wang‐Allison p‐values for sex stratified analysis were calculated only for τ = 50th percentile.

Abbreviations: CD, control diet; GHRH, growth hormone‐releasing hormone; HFD, high fat diet; KO, knock out; WT, wild‐type.

Cox proportional hazard analysis of pooled sex WT mice revealed a significantly greater hazard ratio of 2.032 resulted from HFD feeding, relative to the CD‐fed WT mice (*p* = 0.0028; Table 1). When the sexes were analyzed separately, no differences were observed between the male diet groups while the female WT mice fed HFD displayed a significantly greater hazard ratio of 3.988 relative to the CD‐fed females (*p* < 0.0001; Table 1). When the sexes were pooled for GHRH‐KO mice, the group switched to HFD displayed a significantly greater hazard ratio of 1.61 relative to the CD‐fed group (*p* = 0.0217; Table 1). These differences disappeared when the sexes were analyzed separately, and while a trend for an increased hazard ratio was observed for GHRH‐KO males fed HFD neither the male nor female GHRH‐KO mice fed HFD displayed significantly greater hazard ratios compared to their sex‐matched CD‐fed counterparts (Table 1). When GHRH‐KO and WT mice fed HFD were compared by Cox proportional hazard, GHRH‐KO mice displayed significantly reduced hazard ratios of 0.297 for pooled sexes, 0.236 for males, and 0.383 for females relative to WT mice fed HFD (*p* < 0.0001, *p* = 0.0028, and *p* < 0.0001 respectively; Table 1). Linear regression models were also applied to assess lifespan, and the results were consistent with the Cox proportional hazard results (Table 1).

**FIGURE 2:**
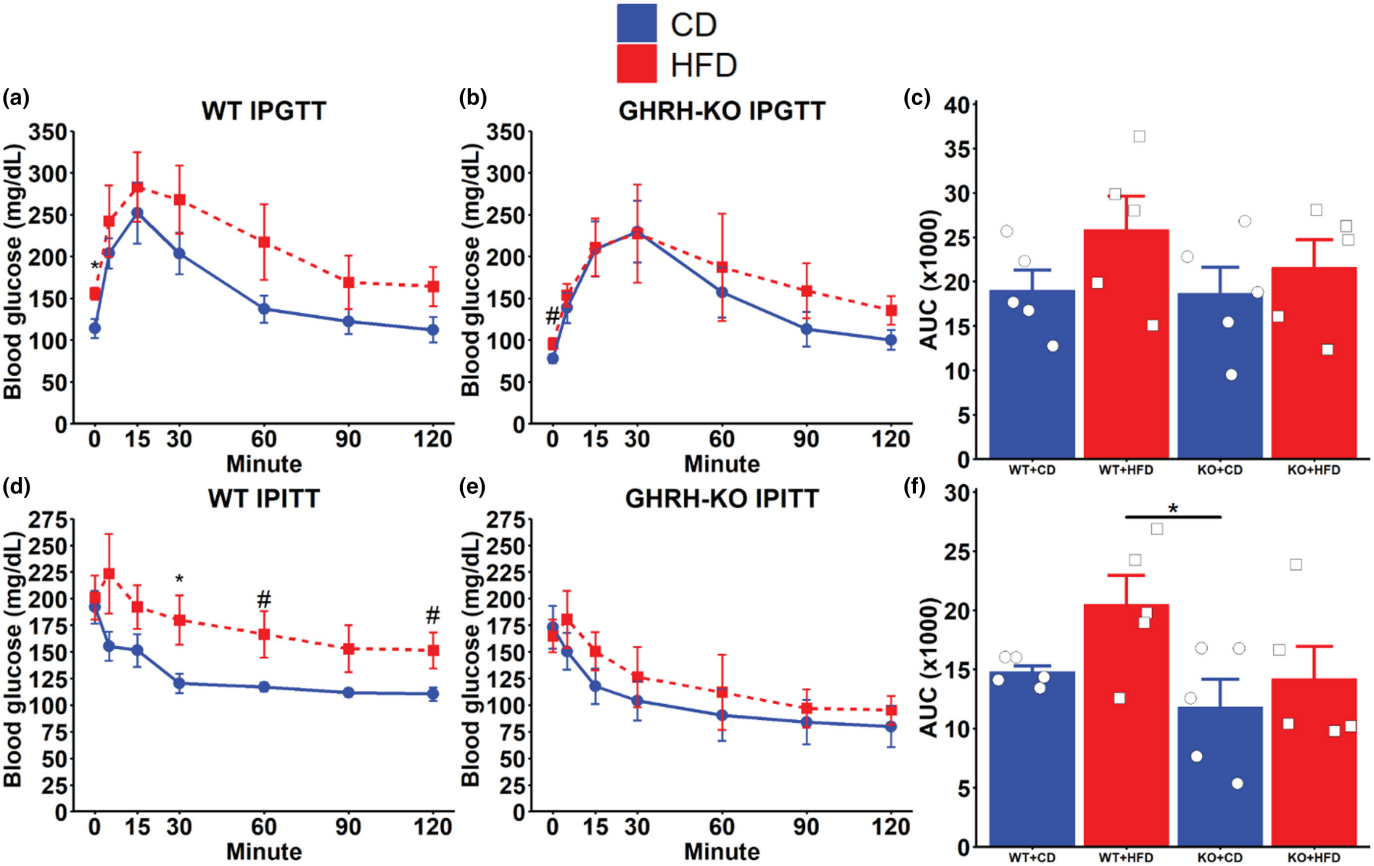
Glucose and insulin tolerance tests. Glycemic response to an intraperitoneal (IP) glucose challenge in female GHRH‐KO (a) and wild‐type (WT) (b) mice after 2.5 months of dietary intervention. Area under the curve (AUC) analysis of the glucose tolerance tests (c). Glycemic response to an IP insulin challenge in female GHRH‐KO (d) and WT (e) mice after 2.5 months of dietary intervention. Area under the curve analyses of the insulin tolerance tests (f). Data presented as mean ± SEM, with points representing individual mice. #*p* < 0.1; *p < 0.05 by Student's *t* test. *N* = 5 for all groups.

Expectedly, we observed reduced bodyweight in GHRH‐KO mice and elevations in bodyweight following 2.5 months of dietary intervention (Figure S1A,B). To assess glucose homeostasis in these mice, we carried out intraperitoneal (IP) glucose and IP insulin tolerance tests after 2.5 months of dietary intervention in females. WT mice fed HFD displayed significantly (*p* = 0.0148) elevated fasting glycemia (Minute 0, immediately before IP glucose challenge) compared to their CD‐fed counterparts and greater mean blood glucose levels at all time points following glucose challenge, however, none reached statistical significance (Figure 2a). GHRH‐KO mice fed HFD displayed a trend for elevated fasting glycemia (*p* = 0.0703), and nearly identical average blood glucose compared to the CD‐fed GHRH‐KO mice through 30 min following a glucose challenge. The HFD‐fed GHRH‐KO mice displayed greater average blood glucose at 60, 90, and 120 min following a glucose challenge; however, these elevations were not statistically significant (Figure 2b). Analysis of the area under the curve (AUC) for the glucose tolerance tests revealed no statistically significant differences between any of the groups examined (Figure 2c). IP insulin tolerance testing showed significantly (*p* = 0.0434) elevated glycemia in the HFD‐fed WT mice at 30 min following an insulin challenge, and trends for elevated glycemia at 60 (*p* = 0.0564) and 120 (*p* = 0.0516) minutes following an insulin challenge compared to CD‐fed WT mice (Figure 2d). In GHRH‐KO mice fed HFD mean glycemia was slightly elevated over CD‐fed GHRH‐KO mice at all time points observed after an insulin challenge; however, none of these reached statistical significance (Figure 2e). AUC analysis for the insulin tolerance tests revealed a statistically significant reduction in AUC between HFD fed WT mice and CD‐fed GHRH‐KO mice (*p* = 0.0340); however, comparisons of all other groups failed to reach statistical significance (Figure 2f).

**FIGURE 3:**
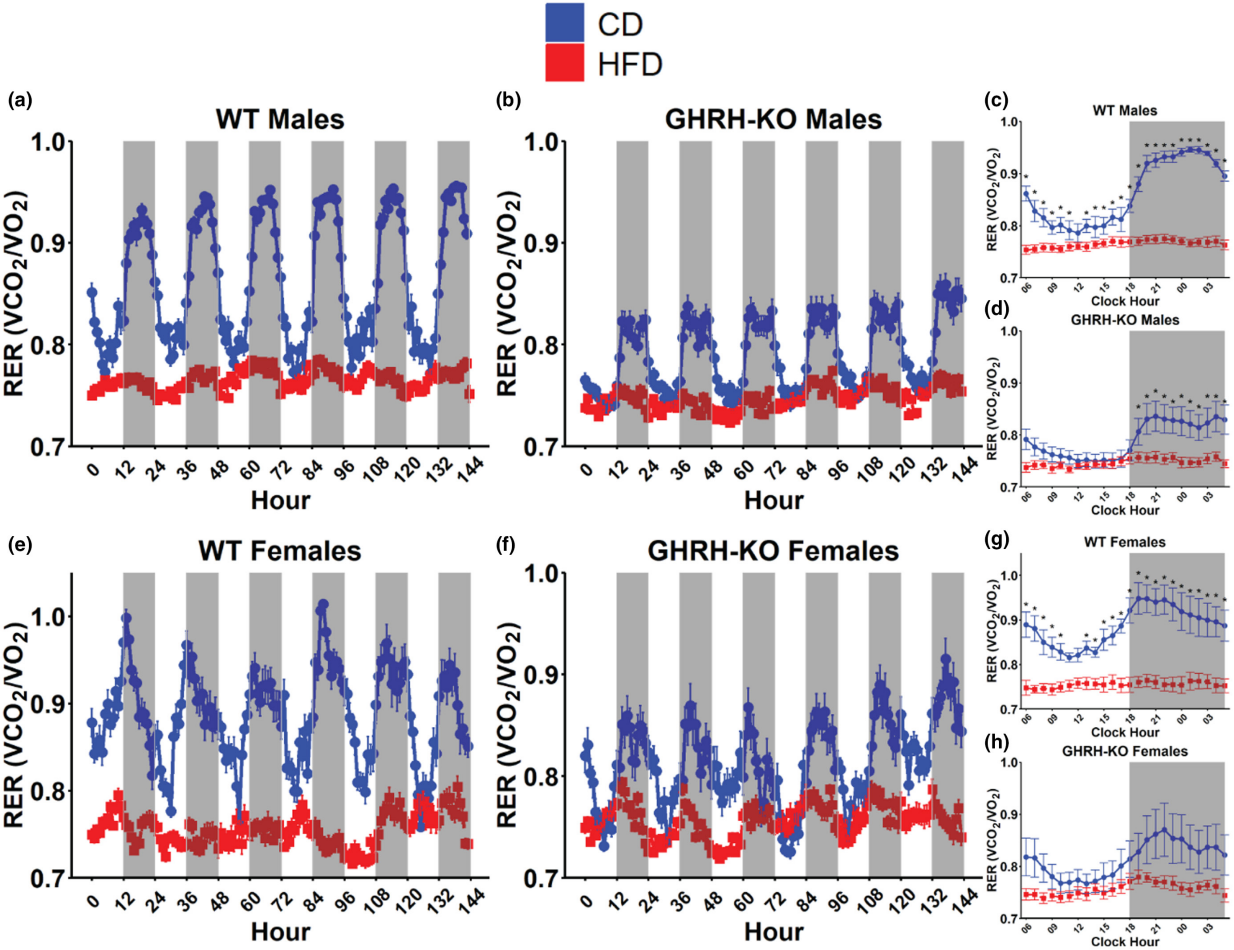
Growth hormone‐releasing hormone (GHRH) deletion or high‐fat diet reduces respiratory exchange ratio (RER). RER (calculated as VCO_2_/VO_2_) for male wild‐type (WT) (a) and male GHRH‐knockout (KO) (b) mice over the 6‐day indirect calorimetry data collection period. Pairwise comparisons for mean RER at each hour in a 24‐h day in WT males (c) and GHRH‐KO males (d). RER for female WT (e) and female GHRH‐KO (f) mice over the 6‐day indirect calorimetry data collection period. Pairwise comparisons for mean RER at each hour in a 24‐h day in WT females (g) and GHRH‐KO females (h). Shaded regions indicate dark cycles. Data presented as mean ± SEM. Statistical significance assessed by repeated measure two‐way ANOVA; *p < 0.05 as determined by pairwise comparisons with the Benjamini–Hochberg false discovery rate correction applied. *N* = 4–9 (males), *N* = 5–6 (females).

To gauge nutrient utilization in these mice following dietary intervention, we collected indirect calorimetry readings from these mice across a continuous 6‐day period. The respiratory exchange ratio (RER; calculated as VCO_2_/VO_2_) is a unitless ratio that varies inversely with fatty acid oxidation (Lusk, 1919). WT male mice fed HFD displayed notably reduced RER during both light and dark cycles compared to WT males fed CD (Figure 3a) while male GHRH‐KO mice fed HFD displayed lower RER during only the dark cycle (Figure 3b). When the mean RER for each hour in a single 24‐h period during the indirect calorimetry session was compared between these groups, WT males fed HFD displayed significantly lower RER at all hours (Figure 3c) while the differences were only significant during the dark cycle for HFD fed GHRH‐KO males (Figure 3d). Similar to the male mice, WT females fed HFD displayed reductions in RER relative to the WT females fed CD (Figure 3e). Again like the male mice, GHRH‐KO females fed HFD displayed reduced RER during only the dark cycle, while no appreciable differences in light cycle RER were observed (Figure 3f). When the average hourly RER was compared between the dietary treatments, WT females fed HFD displayed significantly lower RER compared to WT females fed CD at all hours (Figure 3g). While the mean RER was lower for the GHRH‐KO females fed HFD, these differences did not reach statistical significance after correction for multiple comparisons (Figure 3h). When the mean RER was evaluated for all groups, male GHRH‐KO mice fed CD displayed significantly lower RER during all cycles compared to WT males fed CD, while no differences in RER were observed between male GHRH‐KO and WT mice fed HFD (Figure S2A,B). A similar pattern was seen in female mice, with GHRH‐KO females fed CD showing a trend for lower RER during the light cycles; however, this did not reach statistical significance and no change between CD‐fed WT and GHRH‐KO mice were observed (Figure S2C,D), consistent with our previous report (Icyuz et al., 2020).

**FIGURE 4:**
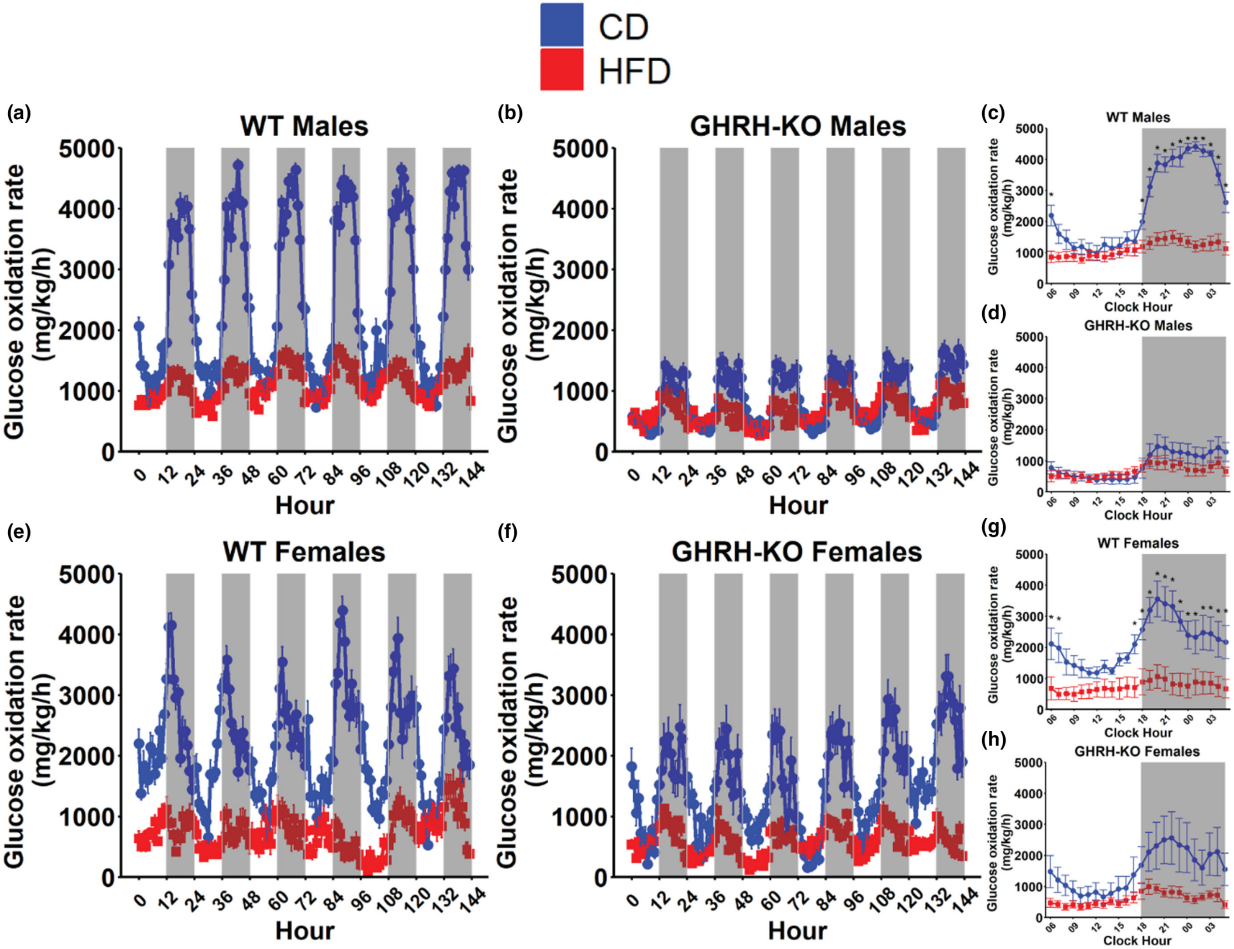
High‐fat diet induced changes in glucose oxidation rate. Body weight normalized glucose oxidation rate, calculated as 4.57 × VCO_2_–3.23 × VO_2_, for male wild‐type (WT) (a) and male growth hormone‐releasing hormone‐knockout (GHRH‐KO) (b) mice over the 6‐day indirect calorimetry data collection period. Pairwise comparisons for mean bodyweight normalized glucose oxidation rate at each hour in a 24‐h day in WT males (c) and GHRH‐KO males (d). Bodyweight normalized glucose oxidation rate for female WT (e) and female GHRH‐KO (f) mice over the 6‐day indirect calorimetry data collection period. Pairwise comparisons for mean bodyweight normalized glucose oxidation rate at each hour in a 24‐h day in WT females (g) and GHRH‐KO females (h). Shaded regions indicate dark cycles. Data presented as mean ± SEM. Statistical significance assessed by repeated measure two‐way ANOVA; *p < 0.05 as determined by pairwise comparisons with the Benjamini–Hochberg false discovery rate correction applied. *N* = 4–9 (males), *N* = 5–6 (females).

We calculated the glucose oxidation rate as previously published (Franczyk et al., 2021; Lasher & Sun, 2023) to assess glucose utilization more completely. WT male mice fed HFD displayed notably reduced bodyweight normalized glucose oxidation during dark cycles compared to WT males fed CD (Figure 4a) while male GHRH‐KO mice fed HFD displayed no appreciable differences in bodyweight normalized glucose oxidation rates (Figure 4b). When the mean bodyweight normalized glucose oxidation rate for each hour in a single 24‐h period during the indirect calorimetry session was compared, WT males fed HFD displayed significantly reduced glucose oxidation during the first hour of the light cycle and at every hour during the dark cycle compared to CD‐fed WT males (Figure 4c). No differences were detected between GHRH‐KO males fed HFD or CD (Figure 4d). WT female mice fed HFD displayed lower bodyweight normalized glucose oxidation, especially during dark cycles, compared to CD‐fed WT females (Figure 4e). GHRH‐KO females fed HFD displayed lower bodyweight normalized glucose oxidation compared to CD‐fed GHRH‐KO females (Figure 4f), although not to the extent seen in WT females. Hourly comparisons of bodyweight normalized glucose oxidation revealed significant reductions in glucose oxidation during the first two and last hours of the light cycle as well as the entire dark cycle for HFD‐fed WT females (Figure 4g). While the glucose oxidation rate was consistently reduced in GHRH‐KO females fed HFD, we did not observe any statistically significant differences after correction for multiple comparisons (Figure 4h). Comparing the mean bodyweight normalized glucose oxidation rates between all groups revealed that in male mice, light cycle mean glucose oxidation was lower in GHRH‐KO mice, but HFD did not significantly affect this (Figure S3A). During the dark cycle, HFD significantly reduced the WT mean glucose oxidation but not the GHRH‐KO glucose oxidation (Figure S3B). In female mice, we detected significant reductions in mean glucose oxidation between WT mice fed CD and GHRH‐KO mice fed HFD, similar to males, and in HFD fed WT mice during both cycles; however, this was not observed in GHRH‐KO mice (Figure S3C,D).

**FIGURE 5:**
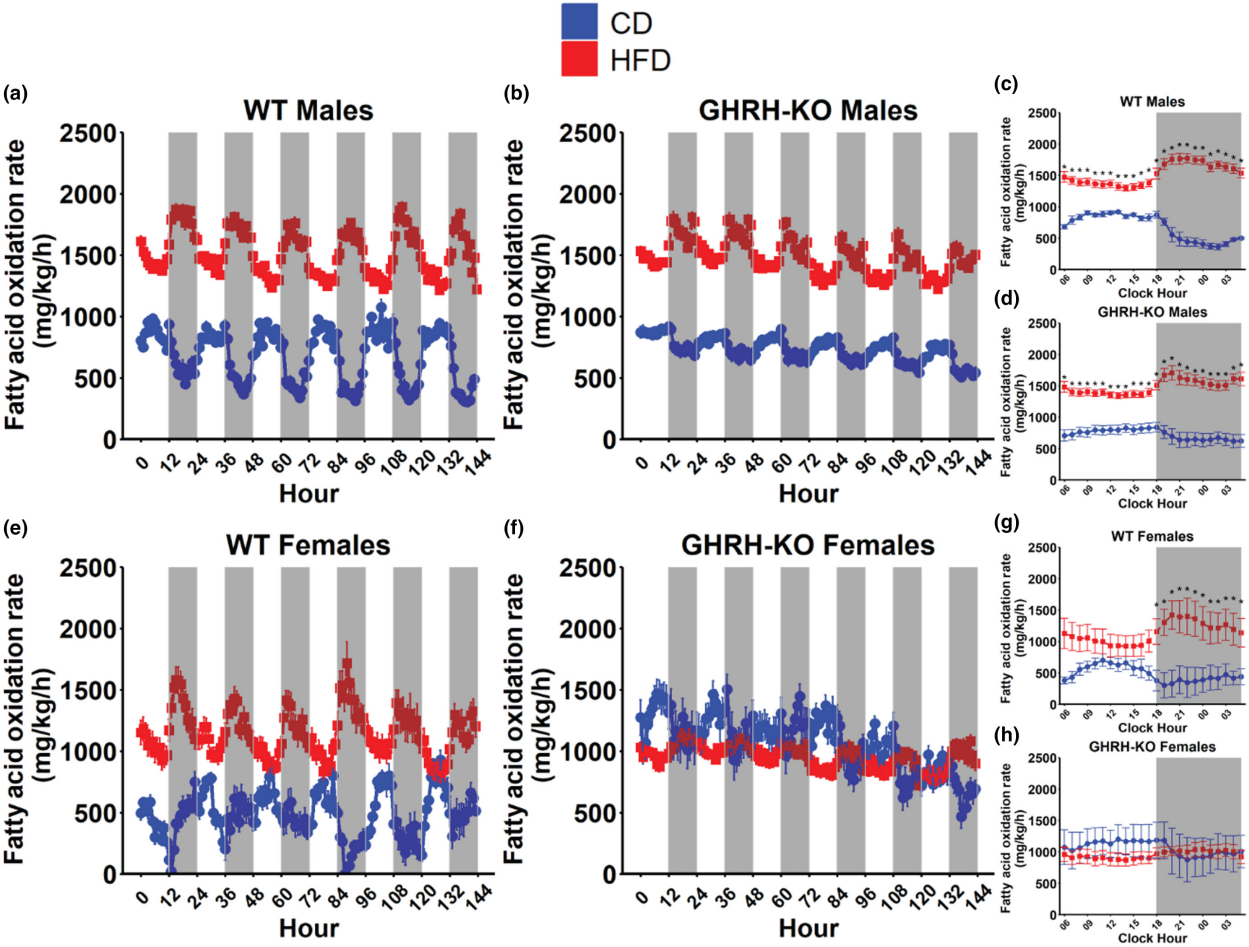
High‐fat diet induced changes in fatty acid oxidation. Body weight normalized fatty acid oxidation rate, calculated as 1.69 × VO_2_–1.69 × VCO_2_, for male wild‐type (WT) (a) and male GHRH‐KO (**b**) mice over the 6‐day indirect calorimetry data collection period. Pairwise comparisons for mean bodyweight normalized fatty acid oxidation rate at each hour in a 24‐h day in WT males (**c**) and growth hormone‐releasing hormone‐knockout (GHRH‐KO) males (d). Bodyweight normalized fatty acid oxidation rate for female WT (e) and female GHRH‐KO (f) mice over the 6‐day indirect calorimetry data collection period. Pairwise comparisons for mean bodyweight normalized fatty acid oxidation rate at each hour in a 24‐h day in WT females (g) and GHRH‐KO females (h). Shaded regions indicate dark cycles. Data presented as mean ± SEM. Statistical significance assessed by repeated measure two‐way ANOVA; *p < 0.05 as determined by pairwise comparisons with the Benjamini–Hochberg false discovery rate correction applied. *N* = 4–9 (males), *N* = 5–6 (females).

We also investigated fat metabolism in these animals by calculating the rate of fatty acid oxidation (Franczyk et al., 2021, Lasher & Sun, 2023). Both WT males and GHRH‐KO males fed HFD displayed marked elevations in bodyweight normalized fatty acid oxidation rates (Figure 5a,b). When the mean bodyweight normalized fatty acid oxidation rates for each hour in a single 24‐h period during the indirect calorimetry session was compared between these groups, both WT and GHRH‐KO males fed HFD displayed significantly elevated fatty acid oxidation rates compared to their genotype‐matched CD‐fed controls (Figure 5c,d). WT females fed HFD displayed comparable bodyweight normalized fatty acid oxidation during light cycles but notable elevations during the dark cycles (Figure 5e). GHRH‐KO females fed HFD did not display appreciable differences in bodyweight normalized fatty acid oxidation (Figure 5f). When hourly mean bodyweight normalized fatty acid oxidation rates were compared, WT females fed HFD displayed significantly elevated fatty acid oxidation during the dark cycle (Figure 5g) while no differences were observed in GHRH‐KO mice fed either diet. (Figure 5h). Also noteworthy were the shifts from the light cycle to the dark cycle in this metric, with all CD groups showing reductions in fatty acid oxidation from light to dark cycle, while the inverse was true in the HFD‐fed groups. When mean bodyweight normalized fatty acid oxidation rates between all groups were compared, we observed that in males HFD significantly elevated mean fatty acid oxidation for both genotypes in light and dark cycles and that fatty acid oxidation was comparable between genotypes for CD or HFD (Figure S4A,B). In females light cycle fatty acid oxidation was elevated in GHRH‐KO mice fed HFD compared to CD‐fed WT mice, and dark cycle fatty acid oxidation was significantly elevated in HFD‐fed WT and GHRH‐KO mice compared to CD‐fed WT mice (Figure S4C,D).

**FIGURE 6:**
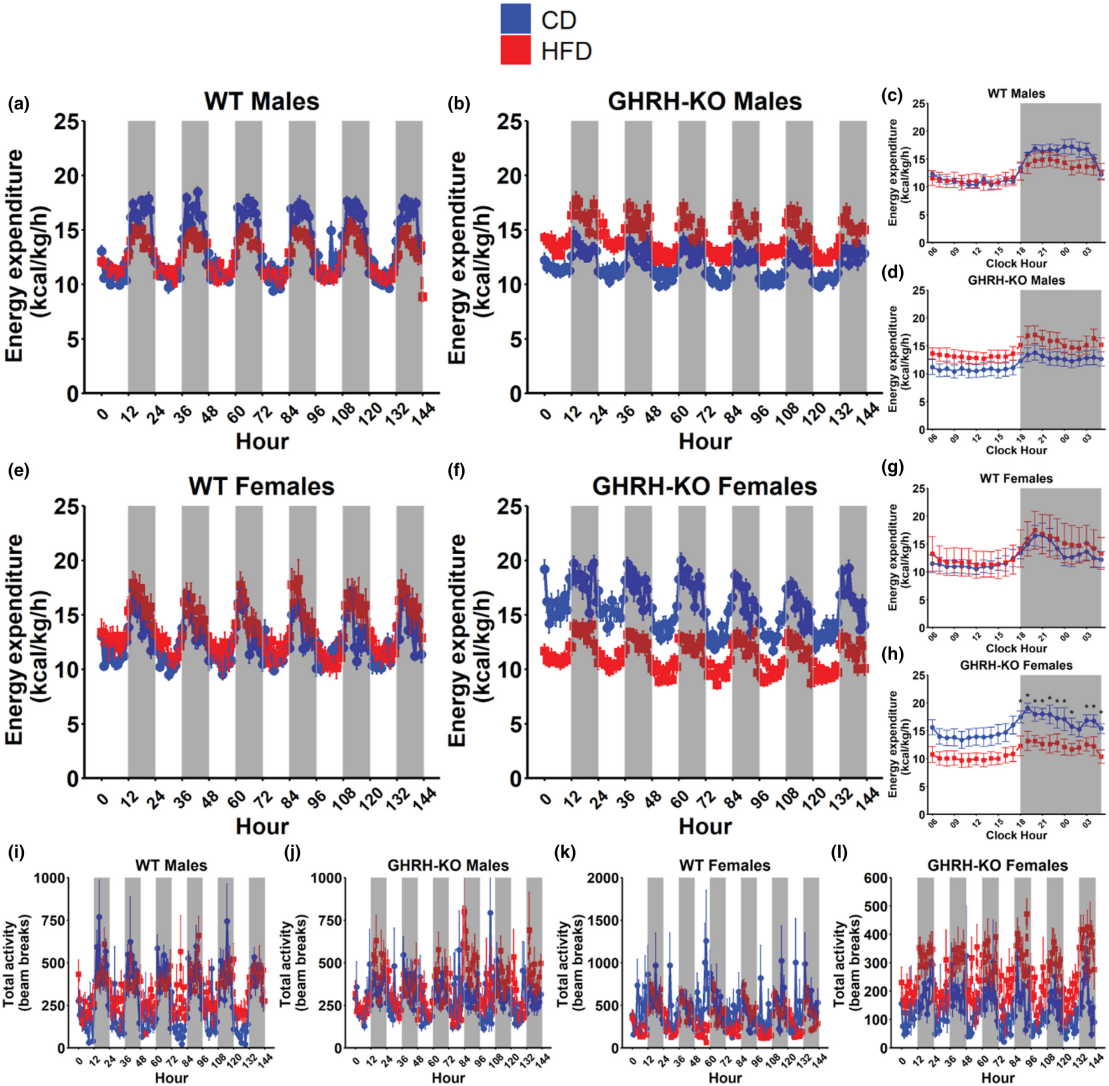
High‐fat diet induced changes in rate of energy expenditure. Body weight normalized energy expenditure, calculated as VO_2_ × (3.815 + 1.232 × RER), for male wild‐type (WT) (a) and male growth hormone‐releasing hormone‐knockout (GHRH‐KO) (b) mice over the 6‐day indirect calorimetry data collection period. Pairwise comparisons for mean bodyweight normalized energy expenditure at each hour in a 24‐h day in WT males (c) and GHRH‐KO males (d). Bodyweight normalized energy expenditure for female WT (e) and female GHRH‐KO (f) mice over the 6‐day indirect calorimetry data collection period. Pairwise comparisons for mean bodyweight normalized energy expenditure at each hour in a 24‐h day in WT females (g) and GHRH‐KO females (h). Total activity, measured as the number of beam breaks across the indirect calorimetry data collection period, for WT males (i), GHRH‐KO males (j), WT females (k), and GHRH‐KO females (l). Shaded regions indicate dark cycles. Data presented as mean ± SEM. Statistical significance assessed by repeated measure two‐way ANOVA; *p < 0.05 as determined by pairwise comparisons with the Benjamini–Hochberg false discovery rate correction applied. *N* = 4–9 (males), *N* = 5–6 (females).

We calculated energy expenditure in our mice, as described in the methods section, to determine the impact of HFD on metabolic rate. WT males fed HFD or CD displayed comparable bodyweight normalized energy expenditure (Figure 6a). Male GHRH‐KO mice fed HFD displayed marginally elevated bodyweight normalized energy expenditure over CD‐fed GHRH‐KO males (Figure 6b). When the mean bodyweight normalized energy expenditure rates for each hour in a single 24‐h period during the indirect calorimetry session was compared between these groups, no differences were detected in WT males (Figure 6c) or GHRH‐KO males (Figure 6d) in either diet group. WT females fed HFD displayed comparable bodyweight normalized energy expenditure to CD‐fed WT females (Figure 5e), but GHRH‐KO females fed HFD displayed notably lower bodyweight normalized energy expenditure compared to CD‐fed GHRH‐KO females (Figure 6f). Hourly comparisons of bodyweight normalized energy expenditure revealed no differences between WT females fed CD or HFD (Figure 6g), but significant reductions in the bodyweight normalized energy expenditure of HFD fed GHRH‐KO females were detected for each hour of the dark cycle (Figure 6h). We also quantified mouse cage activity during the indirect calorimetry experiments, measured as beam breaks detected in the infrared beam grid. No significant differences were observed in this metric (Figure 6i–l), but GHRH‐KO females fed HFD displayed marginally elevated cage activity compared to CD‐fed GHRH‐KO females (Figure 6l) suggesting that cage activity was not a cause for their difference in energy expenditure. No differences were detected when mean bodyweight normalized energy expenditure was compared for all groups (Figure S5).

## 3 DISCUSSION

Investigating the response of long‐lived animal models to dietary stress is critical for understanding the biological control of lifespan and uncovering targets for extending longevity. In this study, we applied HFD stress during the mid (9‐months‐old) and late (18‐months‐old) stages of adulthood in a well‐established, long‐lived mouse model, the first study (to the best of our knowledge) to employ this protocol and feature female mice, to examine the ultimate effect of high dietary fat intake through advanced age in long‐lived mice. We show that the CRISPR/Cas9 GHRH‐KO mutant mice display an extended lifespan over WT mice when subjected to HFD feeding initiated in adulthood, consistent with our previous of extended lifespan in GHRH‐KO mice generated using homologous recombination maintained under standard conditions (Icyuz et al., 2020; Sun et al., 2013; Zhang et al., 2020). Female mice, both WT and GHRH‐KO, displayed markedly reduced lifespans in response to HFD while the lifespans of GHRH‐KO males or WT males were not reduced in response to HFD. To examine the metabolic consequences of HFD feeding in these mice through advanced age, we employed glucose tolerance testing, insulin tolerance testing, and indirect calorimetry on GHRH‐KO and WT mice fed HFD from 18 to 20.5 months of age. GHRH‐KO mice subjected to HFD displayed enhanced insulin sensitivity and resistance to the decreased glucose oxidation observed in WT mice. Our assessment of fatty acid oxidation revealed that male WT and GHRH‐KO mice display remarkably elevated fatty acid oxidation under HFD stress, and while female WT mice display this same elevation, GHRH‐KO females fed HFD displayed no change in this metric compared to CD‐fed genotype/sex‐matched controls. Analysis of metabolic rate in these mice shows that while WT males, WT females, and GHRH‐KO males displayed no changes in energy expenditure under HFD feeding, GHRH‐KO females displayed reduced energy expenditure during the dark cycle. Taken together, our data demonstrate that growth hormone deficiency, mediated by genetic deletion of GHRH, grants extended survival under the stress of high dietary fat intake through adulthood and that notable differences exist in the metabolism of these mice when analyzed in‐vivo.

Comparable to our observations, the hypopituitary Ames dwarf mice retain insulin sensitivity when fed HFD in adulthood, which has been hypothesized to be the result of reduced levels of circulating inflammatory cytokines (Hill et al., 2016). GH receptor knockout mice and pregnancy‐associated plasma protein‐A (PAPP‐A) knockout mice, deficient in insulin‐like growth factor 1 (a GH‐associated peptide), also display reductions in inflammatory cytokine levels despite disproportionately high adipose accumulation when fed high‐calorie diets (Baquedano et al., 2014; Hill et al., 2015). These effects may be partly mediated by the unique adipose tissue biology of GH‐deficient mice. When white adipose was transplanted from HFD‐fed Ames dwarf mice to HFD‐fed WT mice, improvements in insulin sensitivity and reduced IL‐6 levels were reported in the WT mice (Hill et al., 2016). The enhanced lifespan of our GHRH‐KO mice through high‐fat feeding is consistent with these reports as reduced inflammation and enhanced insulin sensitivity have been associated with increased lifespan (Bartke, 2021; Marín‐Aguilar et al., 2020; Spadaro et al., 2016).

HFD feeding initiated in 6–12‐month‐old mice has previously been shown to shorten lifespan in laboratory mice (Minor et al., 2011; Mitchell et al., 2014), consistent with our present report where we initiated HFD in 9‐month or 18‐month old mice. It is worth noting that the present body of literature features C57BL/6J mice with comparatively fewer studies exploring the effects of HFD stress across different genetic backgrounds. In the above‐mentioned works, the median lifespan was reduced by 30–40 weeks in HFD‐treated mice relative to controls. Recently Shi and colleagues reported that the lifespan of HFD‐fed male Wistar rats, an outbred strain, was reduced by 11 weeks compared to CD‐fed controls (Mitchell et al., 2014). While this certainly indicates lifespan was indeed reduced by HFD, the scale of this effect is between one‐third and one‐fourth that of what has been reported in mice. Together, these strongly indicate that the effects of high dietary fat intake on lifespan are not linear. Calorie restriction, which has been more extensively studied in the context of longevity, is known to have differential effects on longevity with both the mouse's sex and genetic background heavily influencing the lifespan response to calorie restriction (Liao et al., 2010). Previous work has shown that female mice display greater increases in lifespan from mild calorie restriction (Mitchell et al., 2016), which may suggest that female mouse lifespan is susceptible to dietary intervention. Consistent with this paradigm, we have previously reported that calorie restriction in GHRH‐KO mice maintained on a mixed C57BL/6J × 129SV genetic background has a more dramatic lifespan‐extending effect in females rather than males (Sun et al., 2013). We hypothesize that the influence of HFD on lifespan, like calorie restriction, is dependent on the interaction of sex, genetic background, and the specific protocol of dietary intervention. This could begin to explain why we observed striking differences between the lifespans of HFD and CD females of the same genotype, yet only minor (statistically insignificant) differences were observed for the males. Further research is required to determine if this is the case.

Among the most commonly observed physiological features of mice with disrupted GH signaling is enhanced insulin sensitivity. Other groups have reported more dramatic responses to exogenous insulin in dwarf mice (Dominici et al., 2002), GH knockout mice (List et al., 2019), and GH receptor knockout mice (Liu et al., 2004) in addition to our group's reports of the same phenomenon in GHRH‐KO mice (Zhang et al., 2020) and double mutant mice lacking both the GH receptor and GHRH (Icyuz et al., 2021). Our data here indicates that this enhanced GHRH‐KO mouse insulin sensitivity is retained through high‐fat feeding in later adulthood, while WT mice displayed insulin insensitivity during the HFD intervention. The reproducibility of this feature, coupled with the opposite feature seen in short‐lived GH overexpressing transgenic mice (Dominici et al., 1999; Wolf et al., 1993) has led to the hypothesis that this is one of the driving factors for the lifespan extension observed in mice with disrupted GH signaling (Aguiar‐Oliveira & Bartke, 2019; Bartke & Brown‐Borg, 2004). The phenotype of our GHRH‐KO mice through HFD is consistent with this paradigm. The different adipose biology of GH‐deficient mice likely contributes to this protection, as both brown and white adipose tissue displays greater metabolic activity in these mice, and white adipose removal negatively affects Ames dwarf insulin sensitivity while improving non‐dwarf insulin sensitivity (Darcy et al., 2016; Menon et al., 2014).

Our indirect calorimetry experiments displayed a pattern of resistance to the effects of HFD in GHRH‐KO mice. RER was dramatically reduced in WT mice fed HFD compared to CD‐fed WTs while the male GHRH‐KO mice fed HFD displayed no difference in the light cycle, and no difference was seen in the dark or light cycle for GHRH‐KO mice. RER is known to be lower in mice deficient in GH, indicating a reduced preference for carbohydrate oxidation to meet metabolic energy demands (Icyuz et al., 2020; Sun et al., 2013; Westbrook et al., 2009). In line with this, our GHRH‐KO mice displayed no differences in glucose oxidation in response to HFD, while WT mice displayed noteworthy reductions in glucose oxidation after HFD. In nearly all cases we observed elevated fat oxidation in the HFD‐fed mice, except for female GHRH‐KO mice where no change in this parameter was observed. We hypothesize that the low reliance on glucose oxidation observed in GHRH‐KO mice under standard feeding conditions confers an advantage in the context of high fat intake as this transition is less stressful on the organism. We also show that female GHRH‐KO mice fed HFD were hypometabolic compared to their CD‐fed counterparts, where no difference was observed between the diets in any other group. This contrasts with reports of IGF‐1 deficient females, where the metabolic rate increased during a shorter‐term high‐fat and high‐sucrose diet intervention (Hill et al., 2015). This indicates that the response of mice with defective GH signaling to dietary stress is likely dependent on dietary composition, rather than only calorie excess.

It is important to consider that the energy intake of our animals was not controlled in this study and that this represents a key limitation of our findings. The distinction between dietary stress from a high fat intake and a high energy intake carries physiological relevance, as a recent report has shown that unrestricted HFD feeding reduces lifespan but that when calorie intake was held constant the higher fat intake extends lifespan (Shi et al., 2021). While the rodent studies in this work were restricted to males and dietary intervention was made at a young age, it indicates that the effects of excess energy intake and excess fat intake have differential consequences for lifespan. Future work should uncouple elevated fat intake and elevated energy intake to delineate the consequences of each to better determine the role of GH signaling in the response to each. It is also noteworthy that our overnight fasting protocol could potentially influence the glucose tolerance data, as this protocol has previously been shown to result in significant lean mass and glycogen loss in mice (Ayala et al., 2006) and potentially mask extant differences in glucose tolerance (Andrikopoulos et al., 2008). While all groups were fasted for the same amount of time before glucose tolerance testing, it is conceivable that the physiology of GH‐deficiency may confer an altered glucose homeostasis following the stress of overnight fasting. Future studies investigating glycemia in these mice should take this stress into consideration.

In summary, our study shows that long‐lived GHRH‐KO mice showed greater resistance to the adverse effects of a high‐fat diet. When both GHRH‐KO and WT mice were exposed to a high‐fat diet in mid‐life, GHRH‐KO mice still lived longer. Intriguingly, this high‐fat diet reduced lifespans in both female GHRH‐KO and WT mice, with no significant difference in males of the same genotype. In a separate experiment, when a high‐fat diet was initiated in late life, GHRH‐KO mice displayed improved insulin sensitivity and fewer metabolic alterations compared to WT mice. These results strongly suggest that the negative impacts of a high‐fat diet are, at least in part, associated with the growth hormone signaling pathway.

## 4 METHODS

### 4.1 Mice

Male and female mice with a CRISPR‐Cas9 mediated germline deletion of the gene encoding GHRH have been previously described (Icyuz et al., 2020). These mice were generated on a C57BL/6J genetic background and crossed to WT BALB/cByJ mice. The resultant offspring were mated to generate GHRH‐KO or WT littermates and were maintained on this mixed BALB/cByJ and C57BL/6J genetic background in an effort to increase genetic diversity and fecundity as we have previously done (Icyuz et al., 2020; Zhang et al., 2020). Mice were weaned from their mothers at 21 days old, separated by sex and genotype, and group housed at a density of 5–7 individuals per cage in a specific pathogen‐free facility maintained on a standard 12‐h light and 12‐h dark cycle at 20–23°C. Mice had ad‐libitum access to standard drinking water and rodent chow (NIH‐31 rat and mouse diet, 18% protein, 4% fat) until 9 months old, at which time they were randomly assigned to their diet group of either control diet (“CD”; research diets D12450K, 10% fat) or high‐fat diet (“HFD”; research diets D12492, 60% fat). Mice had ad‐libitum access to their assigned diet until the end of their life and were not subject to any additional experimental manipulation.

A separate cohort of male and female mice was maintained on standard rodent chow until 18 months of age, at which time they were randomly assigned to either CD or HFD for two and a half months before metabolic assessments were carried out, as described below. These mice had ad‐libitum access to their assigned diet, except where they were fasted for experimentation. Animal protocols were approved by the Animal Care and Use Committee of the University of Alabama at Birmingham.

### 4.2 Lifespan analysis

Several statistical methods were performed to assess the effect of dietary intervention on the lifespan of WT and GHRH‐KO mice, with sexes pooled or separated as indicated. Log‐rank tests and Cox proportional hazard survival models were applied to compare overall survival and the hazard of dietary intervention in each group. The impact of dietary intervention on maximal lifespan was assessed using a quantile regression as described by Wang et al. (2004) where the survival between the groups at the 50th, 75th, and 90th percentiles were compared. The small sample size that resulted when the sexes were analyzed separately prevented meaningful analysis using this method, and the upper (75th and 90th) percentiles were only analyzed when the sexes were pooled. Additionally, lifespan was assessed by linear regression models as described previously (Sun et al., 2017).

### 4.3 Metabolic assessments

Glucose tolerance testing (GTT) was carried out in overnight fasted (approximately 18 h) mice. Blood glucose measurements were taken immediately prior (“Minute 0”) to an IP injection of 1 g/kg glucose, and at the time points indicated post‐injection. Insulin tolerance testing was carried out in 4‐h fasted mice. Blood glucose measurements were taken immediately prior (“Minute 0”) to an IP injection of 1 U/kg porcine insulin (Sigma), and at the time points indicated post‐injection. Blood glucose measurements were taken using an AgaMatrix PRESTO handheld glucometer.

Indirect calorimetry experiments were carried out using the comprehensive lab animal monitoring system (CLAMS, Columbus Instruments). This system uses zirconia and infrared sensors to monitor oxygen consumption (VO_2_) and carbon dioxide production (VCO_2_), an electronic balance system to monitor food consumption, and an infrared beam grid to monitor mouse activity. Mice were individually housed in calorimetry chambers for a 6‐day acclimation period where no data was collected, followed by a 6‐day data collection period where VO_2_, VCO_2_, food consumption, and activity data were collected continuously every 9 min per mouse. Respiratory parameters were normalized to total body weight to control for differences that occur due to the size difference between animals. Respiratory exchange ratio (RER) and energy expenditure were calculated as VCO_2_/VO_2_ and VO_2_ × (3.815 + 1.232 × RER), respectively (Icyuz et al., 2020, 2021). Glucose oxidation rate and fatty acid oxidation rate were calculated as 4.57 (VCO_2_)–3.23 (VO_2_) and 1.69 (VO_2_)–1.69 (VCO_2_), respectively (Franczyk et al., 2021; Lasher & Sun, 2023).

### 4.4 Statistical analysis

Lifespan data were analyzed as described above. For metabolic assessments, group means were compared using the two‐tailed student's *t* test. A two‐way repeated measure ANOVA was used to analyze metabolic data with post‐hoc Benjamini–Hochberg corrected comparisons carried out where significant main effects were detected. Means were considered statistically significant at *p* < 0.05. Analyses were carried out and figures were generated using the R programming language.

## AUTHOR CONTRIBUTIONS

The manuscript was primarily written by Joseph Adkins‐Jablonsky and Alexander Tate Lasher. Data collection was a collaborative effort involving Joseph Adkins‐Jablonsky, Alexander Tate Lasher, and Akash Nagarajan. Data analysis was performed by Joseph Adkins‐Jablonsky, Alexander Tate Lasher, and Amit Patki. Liou Y. Sun conceived the study, designed experiments, supervised the overall direction, helped to write the manuscript, and secured funding.

## FUNDING INFORMATION

NIH grants AG082327 and AG057734.

## CONFLICT OF INTEREST STATEMENT

All contributing authors declare no conflict of interest.

## Supporting information


Figure S1.

## Data Availability

All of the data supporting the findings of this study are available from the corresponding author upon reasonable request.
