# Supplementary material for: Growth hormone‐releasing hormone deficiency confers extended lifespan and metabolic resilience during high‐fat feeding in mid and late life
Source: Aging Cell. 2024 Jun 12;23(9):e14238. doi: 10.1111/acel.14238 (PMC11488314; doi:10.1111/acel.14238)
Supplement: Supplementary file 1 — Figure S1. [file ACEL-23-e14238-s001.docx]

Supplemental Figure 1


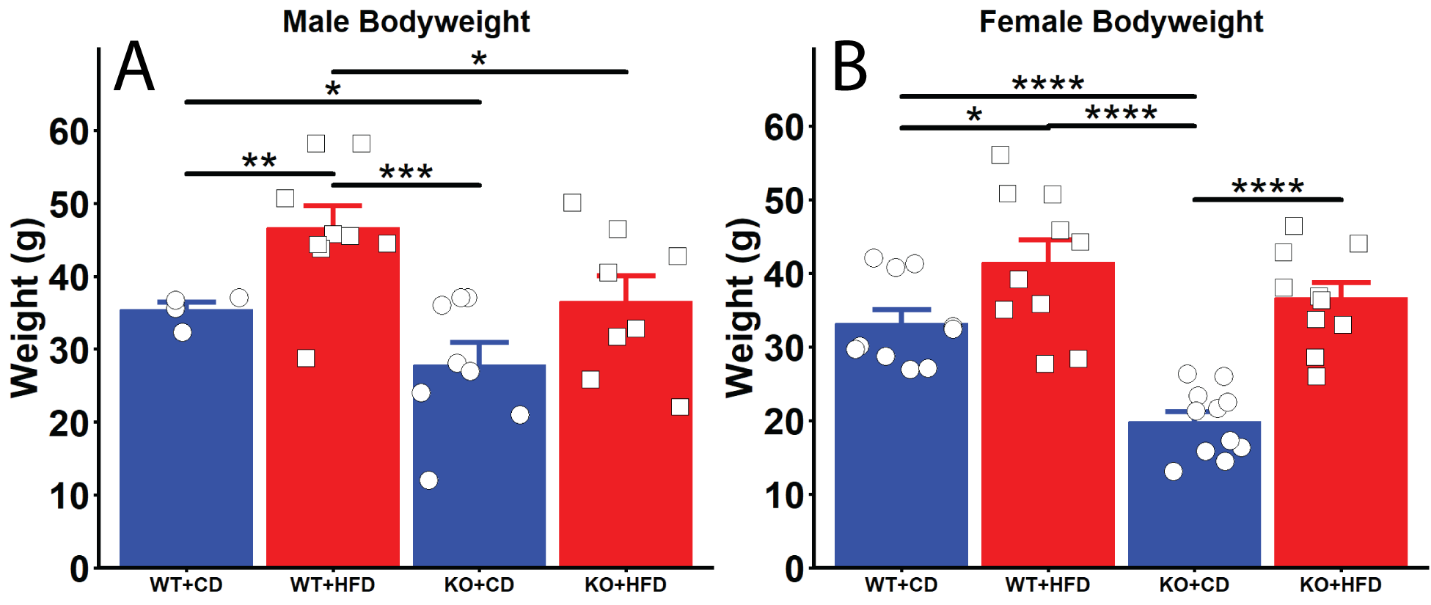


**Supplemental Figure S1.** Bodyweight of WT and GHRH-KO male (**A**) and female (**B**) mice following 2.5 months of dietary intervention. Data presented as mean ± SEM with points representing individual mice. *p<0.05; **p<0.01; ***p<0.001; ****p<0.0001 as determined by a two-tailed t-test. N=4-9 (males), N=10-11 (females).

Supplemental Figure 2

**
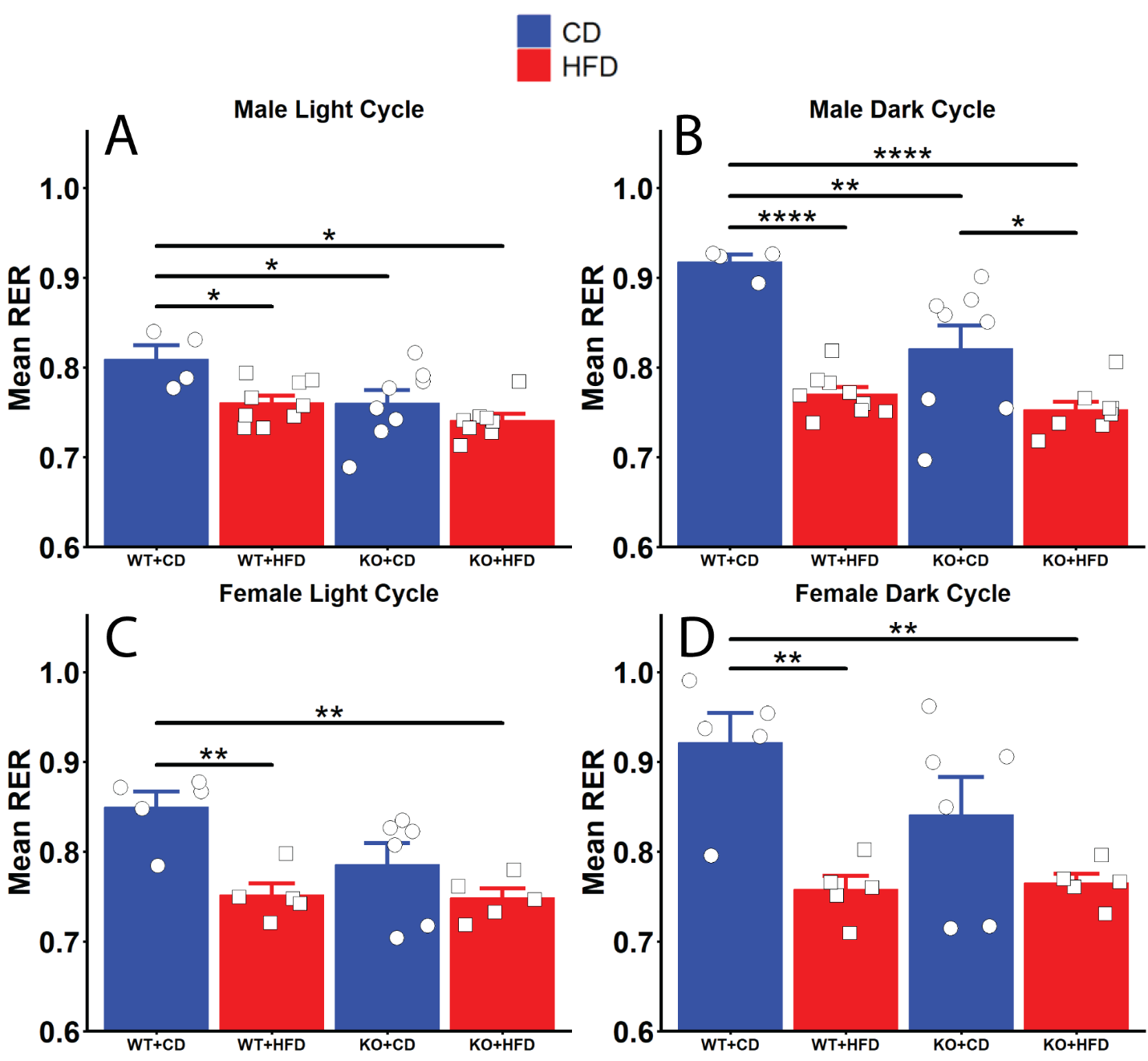
**

**Supplemental Figure S2.** Companion to figure 3: high-fat diet or GHRH deletion reduces RER. Mean RER during the light cycle for control diet or high-fat diet fed male WT and GHRH-KO mice (**A**). Mean RER during the dark cycle for control diet or high-fat diet fed male WT and GHRH-KO mice (**B**). Mean RER during the light cycle for control diet or high-fat diet fed female WT and GHRH-KO mice (**C**). Mean RER during the dark cycle for control diet or high-fat diet fed female WT and GHRH-KO mice (**D**). Data presented as mean ± SEM with points representing individual mice. *p<0.05; **p<0.01; ***p<0.001; ****p<0.0001 as determined by a two-tailed t-test. N=4-9 (males), N=5-6 (females).

Supplemental Figure 3

**
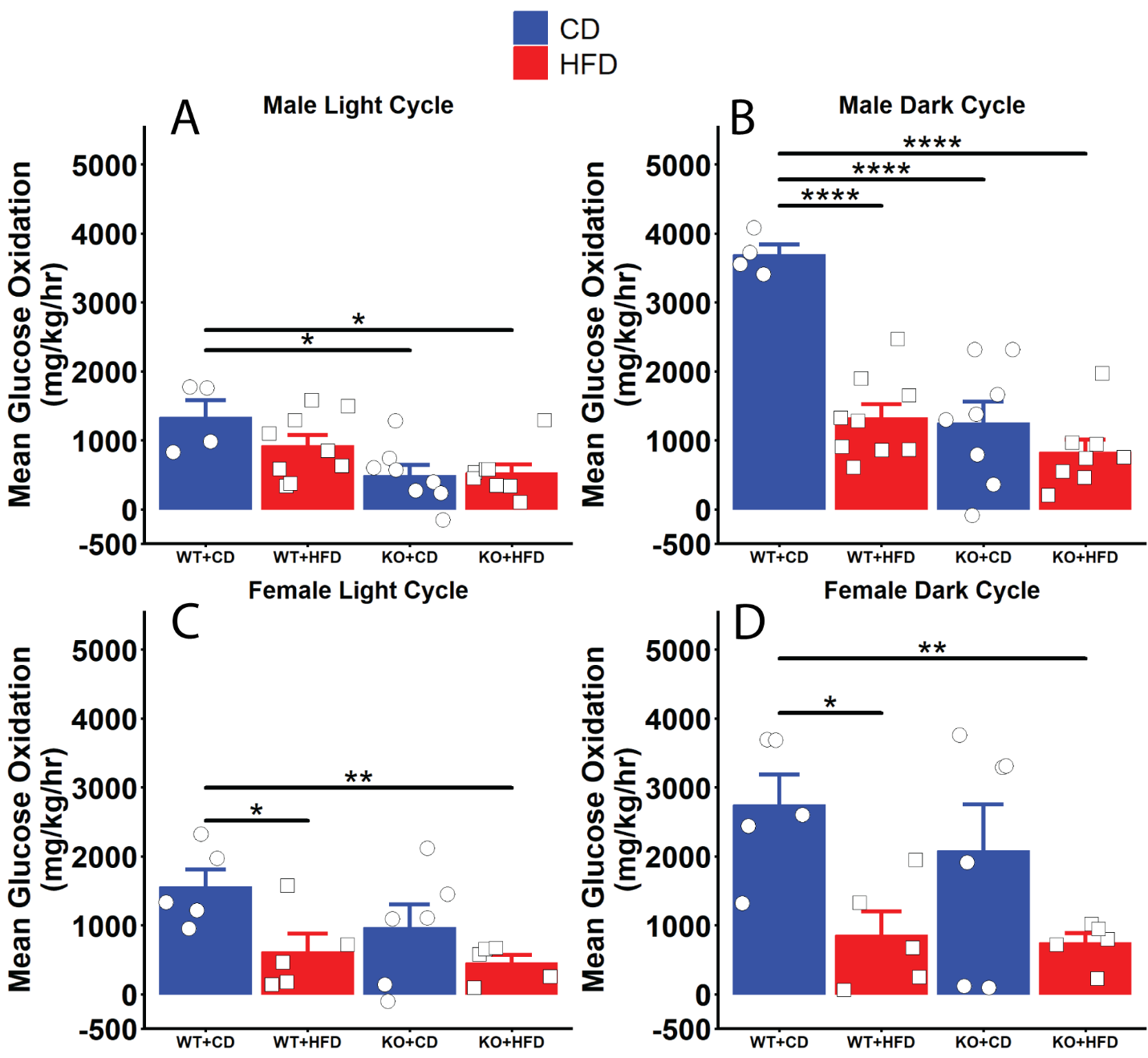
**

**Supplemental Figure S3.** Companion to figure 4: high-fat diet or GHRH deletion reduces glucose oxidation rate. Mean bodyweight normalized glucose oxidation rate during the light cycle for control diet or high-fat diet fed male WT and GHRH-KO mice (**A**). Mean bodyweight normalized glucose oxidation rate during the dark cycle for control diet or high-fat diet fed male WT and GHRH-KO mice (**B**). Mean bodyweight normalized glucose oxidation rate during the light cycle for control diet or high-fat diet fed female WT and GHRH-KO mice (**C**). Mean bodyweight normalized glucose oxidation rate during the dark cycle for control diet or high-fat diet fed female WT and GHRH-KO mice (**D**). Data presented as mean ± SEM with points representing individual mice. *p<0.05; **p<0.01; ***p<0.001; ****p<0.0001 as determined by a two-tailed t-test. N=4-9 (males), N=5-6 (females).

Supplemental Figure 4

**
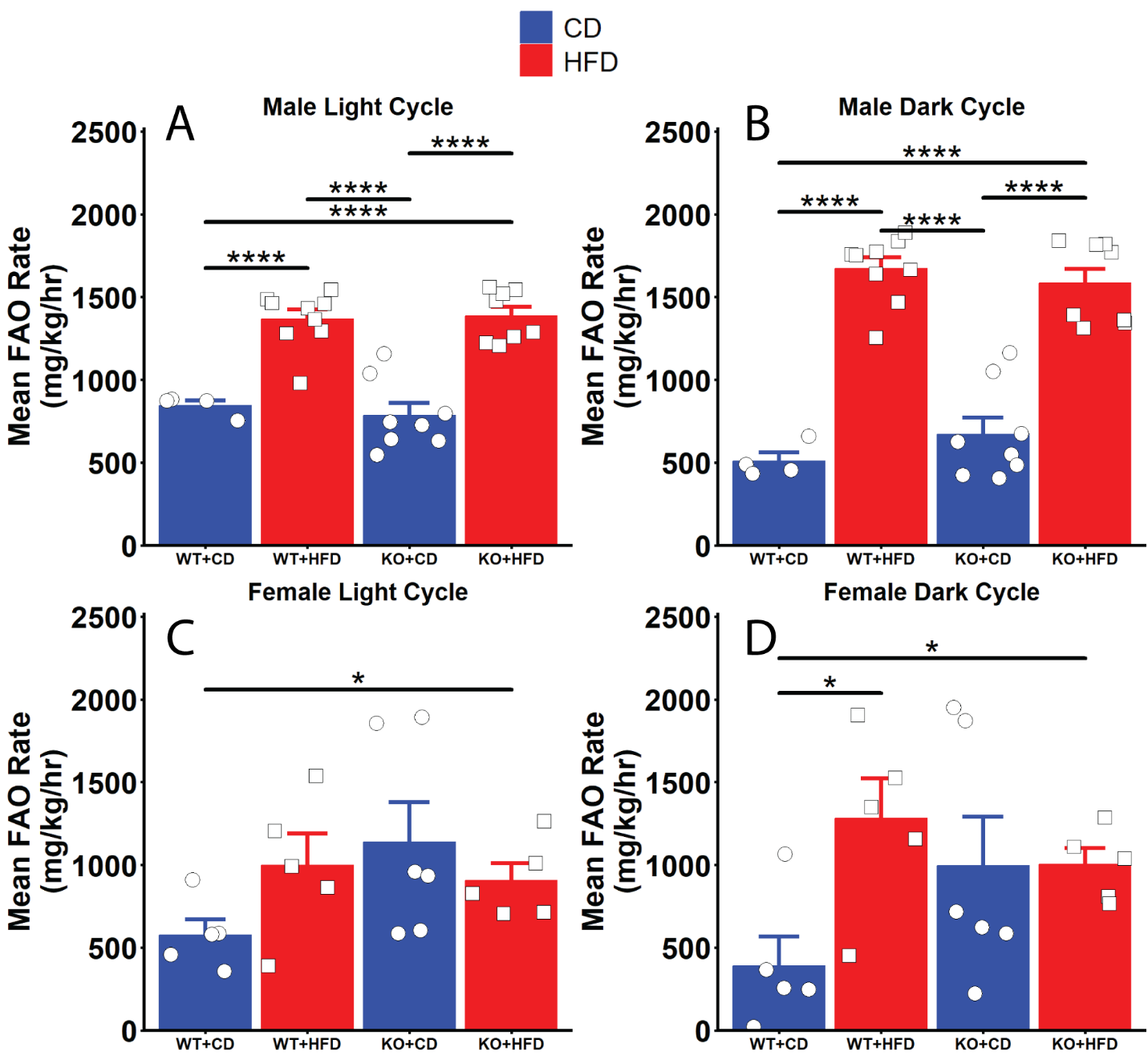
**

**Supplemental Figure S4.** Companion to figure 5: high-fat diet elevates fatty acid oxidation (FAO) rate. Mean bodyweight normalized FAO rate during the light cycle for control diet or high-fat diet fed male WT and GHRH-KO mice (**A**). Mean bodyweight normalized FAO rate during the dark cycle for control diet or high-fat diet fed male WT and GHRH-KO mice (**B**). Mean bodyweight normalized FAO rate during the light cycle for control diet or high-fat diet fed female WT and GHRH-KO mice (**C**). Mean bodyweight normalized FAO rate during the dark cycle for control diet or high-fat diet fed female WT and GHRH-KO mice (**D**). Data presented as mean ± SEM with points representing individual mice. *p<0.05; **p<0.01; ***p<0.001; ****p<0.0001 as determined by a two-tailed t-test. N=4-9 (males), N=5-6 (females).

Supplemental Figure 5

**
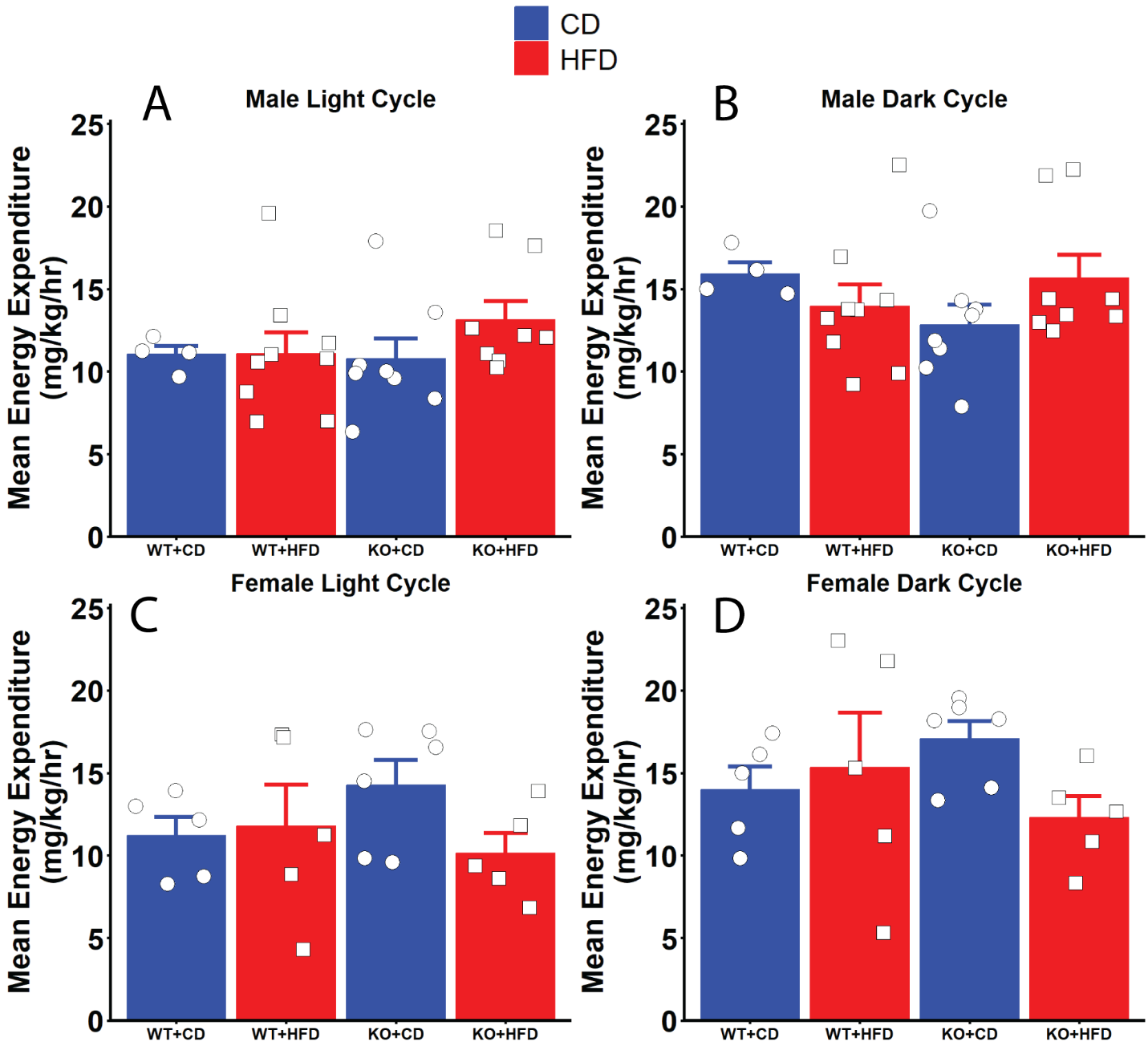
**

**Supplemental Figure S5.** Companion to figure 6: high-fat diet does not alter mean energy expenditure. Mean bodyweight normalized energy expenditure during the light cycle for control diet or high-fat diet fed male WT and GHRH-KO mice (**A**). Mean bodyweight normalized energy expenditure during the dark cycle for control diet or high-fat diet fed male WT and GHRH-KO mice (**B**). Mean bodyweight normalized energy expenditure during the light cycle for control diet or high-fat diet fed female WT and GHRH-KO mice (**C**). Mean bodyweight normalized energy expenditure during the dark cycle for control diet or high-fat diet fed female WT and GHRH-KO mice (**D**). Data presented as mean ± SEM with points representing individual mice. *p<0.05; **p<0.01; ***p<0.001; ****p<0.0001 as determined by a two-tailed t-test. N=4-9 (males), N=5-6 (females).
